# Dietary Patterns, Adherence to the Food-Based Dietary Guidelines, and Ultra-Processed Consumption During the COVID-19 Lockdown in a Sample of Spanish Young Population

**DOI:** 10.3389/fped.2021.702731

**Published:** 2021-10-22

**Authors:** José Francisco López-Gil, Antonio García-Hermoso, Pedro Juan Tárraga-López, Javier Brazo-Sayavera

**Affiliations:** ^1^Departamento de Expresión Plástica, Musical y Dinámica, Facultad de Educación, Universidad de Murcia, San Javier, Spain; ^2^Health and Social Research Center, Universidad de Castilla-La Mancha (UCLM), Cuenca, Spain; ^3^Navarrabiomed, Complejo Hospitalario de Navarra (CHN), Universidad Pública de Navarra (UPNA), Pamplona, Spain; ^4^Escuela de Ciencias de la Actividad Física, el Deporte y la Salud, Universidad de Santiago de Chile (USACH), Santiago, Chile; ^5^Departamento de Ciencias Médicas, Facultad de Medicina, Universidad Castilla-La Mancha (UCLM), Albacete, Spain; ^6^Polo de Desarrollo Universitario EFISAL, Centro Universitario Regional Noreste, Universidad de la República, Rivera, Uruguay; ^7^Department of Sports and Computer Science, Universidad Pablo de Olavide, Seville, Spain

**Keywords:** healthy diet, nutrition, healthy lifestyle, youths, health behavior, social distance

## Abstract

**Purpose:** The aim of this study was to explore the dietary patterns, adherence to Food-Based Dietary Guidelines, and the ultra-processed consumption during the COVID-19 lockdown among a Spanish young population aged 3–17 years.

**Methods:** Parents/legal guardians of preschoolers, children, and adolescents aged 3–17 years were enrolled through social networks. The eating habits were assessed by a Food Propensity Questionnaire applied in the ENALIA (*Encuesta Nacional de Alimentación en la población Infantil y Adolescente*) Spanish survey, which aims to collect food intake information and other data about eating habits on children and adolescents (0–18 years old). Participants were dichotomized following the Food-Based Dietary Guidelines for the Spanish young population offered by the Spanish Society of Community Nutrition. The ultra-processed food score was determined following the principles established in the NOVA classification.

**Results:** Data from 604 children and adolescents were included. An association between age group and the recommendations of snacks (*p* = 0.002), fruits (*p* = 0.010), and diaries (*p* < 0.001) was found. Adolescents showed a lower mean compliance with these guidelines than children (*p* = 0.004) and preschoolers (*p* < 0.001). Similarly, children reported lower Food-Based Dietary Guidelines than preschoolers (*p* = 0.015). Regarding ultra-processed consumption, it was also observed a higher intake in adolescents than in children (*p* = 0.037), as well as in preschoolers (*p* < 0.001).

**Conclusions:** The associations that were found highlight the low proportion of the young population (especially adolescents) meeting the Food-Based Dietary Guidelines and the high consumption of ultra-processed foods during COVID-19 lockdown.

## Introduction

As a result of the current COVID-19 crisis, public health recommendations and Spanish governmental measures have implemented restrictions and lockdown, such as stay-at-home orders, mandatory mask requirements, limitation of the freedom of people movement, suspension of the on-site educational activities (replaced by online educational activities), and/or close of the public establishments (with the exception of those essential) (1). Although these strategies assist to decrease the rate of infection, such restrictions (e.g., increased social distancing) involve adverse consequences by restraining engagement in physical activity, normal day-to-day routines, and access and travel to several ways of exercise (2).

Dietary risk factors are ranked among the principal risk factors for disability and have been identified as responsible for a large proportion of chronic non-communicable diseases worldwide (3). Supporting this notion, holistic and integrated approaches across all sectors and policy areas are needed to deal with the worrying prevalence of non-communicable diseases with an emphasis on primary prevention (4). Similarly, providing assistance based on evidence for healthier lifestyles and dietary patterns could exert an essential role for public health (5). In this sense, food-based dietary guidelines are helpful tools for public health strategies and nutrition policies to encourage healthier eating habits (6, 7).

The World Health Organization has strongly recommended to follow a healthy diet (8) during the periods of lockdown. Thus, this diet should include fruits, legumes, whole grains, vegetables, and healthy fats. In this sense, one study performed by Ammar et al. (9) during the COVID-19 lockdown indicated that this situation alters eating behaviors (eating out of control, an overall greater number of main meals, higher intake of unhealthy food, and more snacking between meals) in a health-compromising direction. Also, other studies performed in Spain showed food consumption changes (i.e., nut, homemade dessert, confectionary, snack, and jelly bean intakes increased) (10), as well as a trend toward greater consumption of healthy foods, lower consumption of foods of less nutritional interest, and an increase in the practice of cooking at home during the lockdown (11).

On the other hand, the intake of ultra-processed foods has been linked with a higher dietary risks of associated non-communicable diseases (12), as well as a less desirable cardiometabolic risk status and a higher risk of both cardiovascular and cerebrovascular diseases, depression, and all-cause mortality (13). Correspondingly, most of the scientific literature on the relationship between intake of ultra-processed foods and adiposity presents a positive direction (i.e., a higher intake leads to increased adiposity) (14). In addition, a recent systematic review with meta-analysis has pointed out the connection between intake of ultra-processed food and metabolic syndrome in youths and dyslipidemia in children (15).

Consequently, it appears reasonable that governments should support policies that promote more efficiently consuming healthy food during periods of lockdown. Although COVID-19 vaccines are being administered in several countries, this fact does not imply that the emergency is almost finished, since we are just beginning a next stage of the pandemic (16). To the best of our knowledge, studies on the effect of the COVID-19 lockdown in food patterns among the young population are still scarce. In this sense, this is the first study which assessed dietary patterns and eating habits during the COVID-19 lockdown among preschoolers, children, and adolescents. Thus, the aim of this study was to explore the diet-related patterns, adherence to Food-Based Dietary Guidelines, and the ultra-processed consumption during the COVID-19 lockdown among the Spanish young population aged 3–17 years. We hypothesize that, during the COVID-19 lockdown, eating habits were inadequate, compliance with nutritional recommendations was low, and consumption of ultra-processed foods was high among the Spanish young population. It is also speculated that these inadequate eating habits were most prevalent in older participants.

## Materials and Methods

### Population Sample and Study Design

Parents/legal guardians of preschoolers/children/adolescents aged 3–17 years were enrolled through social media (Facebook, Instagram, Twitter, and LinkedIn). An online survey was generated and sent by a snowball sampling technique. In this sense, apart from recruiting through social media, we invited researchers from various regions of Spain to disseminate our survey, with the aim of trying to reach a more varied and larger number of participants. To fulfill the online survey, around 15 min were needed. Prior to filling in the online survey, data about the aim of the research were explained and an informed consent was required. Data were collected for 15 days (from March 29 to April 13, 2020). In this period, the entire Spanish population should remain at home (except essential workers) and was only allowed to go out for basic food shopping, healthcare, and some justified exceptions (1). Of the first 720 respondents, 77 participants were excluded since they were under 3 years or over 17 years of age. Furthermore, 41 participants were excluded due to missing information. Finally, data from 604 respondents were incorporated in the final analysis.

In terms of inclusion criteria, only parents/legal guardians of the Spanish young population aged 3–17 years who signed the informed consent were included. Conversely, regarding the exclusion criteria, participants were not enrolled when they did not completely fill out the online survey.

This study was conducted following the Helsinki Declaration for Human Studies and approved by the Ethical Committee of the Universidad Católica de Murcia (UCAM) (code: CE112001). All participants and their parents/legal guardians were informed of the aim of the research, and then a written informed consent was required.

### Procedures

#### General Information

Parents/legal guardians were requested to fulfill the online survey. The initial section informed participants about the study design and aims of the study. Parents'/legal guardians' information about sex and age (calculated from date of birth) of their children, educational level, and socioeconomic status [through the Family Affluence Scale—FAS-III (17)] was required. Information on geographic location was also requested. Similarly, anthropometric information was reported by parents/legal guardians about the minors. Weight was self-declared in kilograms and height in meters. Both the z-score for body mass index and the categorization of excess weight (overweight/obesity) were computed adhering to the World Health Organization standards (18, 19).

#### Food-Based Dietary Guidelines

The eating habits were assessed by a Food Propensity Questionnaire (FPQ) (20) applied in the ENALIA (*Encuesta Nacional de Alimentación en la población Infantil y Adolescente*) Spanish survey, which aims to collect food intake information and other data about dietary patterns on children and adolescents (0–18 years old). A detailed explanation of this survey was published elsewhere (21). Participants were dichotomized following the Food-Based Dietary Guidelines for the Spanish young population offered by the Spanish Society of Community Nutrition (SENC) (22). A detailed explanation about the establishment of the adherence to the Food-Based Dietary Guidelines is shown in Supplementary Table 1.

#### Ultra-Processed Food Score

The ultra-processed food score was determined, following the principles established in NOVA classification (12). The NOVA system classifies all beverages and foods into four groups based on their own nature, purpose, and extent of factory food manufacturing: 1—minimally processed foods or unprocessed; 2—processed cooking ingredients; 3—processed foods; and 4—ultra-processed foods. Group of foods were considered ultra-processed when they contain any formulation made mainly or completely from products derived from additives and foods (i.e., savory or sweet packaged snacks, soft drinks). Thus, 20 groups of foods were considered as ultra-processed food. Due to the lack of a specific score to determine the consumption of these foods, responses were scored as follows: 0—never, 1—one to three times a month; 2—once a week; 3—two or three times a week; 4—four to six times a week; 5—once a day; 6—more than once a day. The final score varied from 0 to 120 points.

#### Covariates

Sex (females or males), socioeconomic status (SES) (high, medium or low) (17), educational level (complete higher education, incomplete higher education, complete secondary education, incomplete secondary education, complete primary education, or incomplete primary education), region (Southern or Northern Spain), BMI (z-score) (18, 19), and physical activity were incorporated as potential covariates. The level of physical activity was according to the next question: “Normally, how many days was your child physically active for a total of at least 60 min?”. The possible options varied from 0 to 7 days weekly. This measure has revealed to have good validity and reliability (23).

### Statistical Analysis

Data were shown as means and standard deviation for continuous variables and frequencies and percentages for categorical variables. Data normality was checked by Kolmogorov-Smirnov tests with Lilliefors correction, and the homogeneity of variances by the Levene test. Kruskal-Wallis H test or one-way ANOVA for three-group comparisons (preschoolers, children, and adolescents), according to the normality assumption. Conversely, Pearson's chi square test was applied to determine associations between qualitative variables. Binary logistic regression analyses were performed to determine the association between meeting the different Food-Based Dietary Guidelines across age groups [preschoolers (aged 3–5), children (aged 6–12), adolescents (13–17)]. Furthermore, analysis of covariance (ANCOVA) was performed to verify the association between means of adherence to the Food-Based Dietary Guidelines across age groups. Preliminary analysis showed no interaction between sexes and both the meeting of the Food-Based Dietary Guidelines (*p* = 0.162) and the ultra-processed food score (*p* = 0.331). For this reason, the analysis was carried out with both sexes together to increase the statistical power. All analyses were conducted with SPSS statistical software version 24 for Windows. The statistical significance level was established at *p* < 0.05.

## Results

Table 1 indicates the descriptive information of participants. The average age was 12.1 (4.6). The sex distribution was similar (50.2% girls). The prevalence of participants with high SES was 22.4%. Moreover, 31.0% of the participants' breadwinner completed higher education. Twenty percent of the sample showed excess weight. Participants from Southern Spain (74.0%) were higher than those from Northern Spain (26.0%). Moreover, the average number of days being physically active was 4.1 (2.3).

**Table 1:** Descriptive information of the Spanish analyzed sample (*n* = 604).

**Variables**	**Preschoolers (*n* = 75; 12.4%)**	**Children(*n* = 208; 34.4%)**	**Adolescents (*n* = 321; 53.1%)**	***p*** **-value for trend**

	**M (SD) /** ***n*** **(%)**	**M (SD) /** ***n*** **(%)**	**M (SD) /** ***n*** **(%)**	
Age (years)	4.2 (0.8)	9.1 (2.0)	15.7 (1.8)	<0.001
Sex				
Males	44 (58.7)	116 (55.8)	141 (43.9)	0.008
Females	31 (41.3)	92 (44.2)	180 (56.1)	
*Anthropometric data*				
Weight (kg)	18.00 (5.54)	32.68 (11.19)	59.11 (13.17)	<0.001
Height (cm)	105.2 (11.8)	134.8 (15.0)	166.4 (9.5)	<0.001
BMI (z-score)	0.55 (2.02)	0.74 (2.08)	1.11 (1.82)	<0.001
*SES*				
High SES, (%)	17 (22.7)	60 (28.8)	58 (18.1)	0.008
*Breadwinner's educational level*				
Complete higher education, (%)	38 (50.7)	96 (46.2)	53 (16.5)	<0.001
*Geographical location*				
Southern Spain, (%)	48 (64.0)	159 (76.4)	239 (74.5)	0.163
*Physical activity*				
Physically active ≥ 60 min (days)	5.1 (2.0)	4.4 (2.3)	3.5 (2.3)	<0.001

*BMI, body mass index; SES, socioeconomic status*.

**Figure 1:**
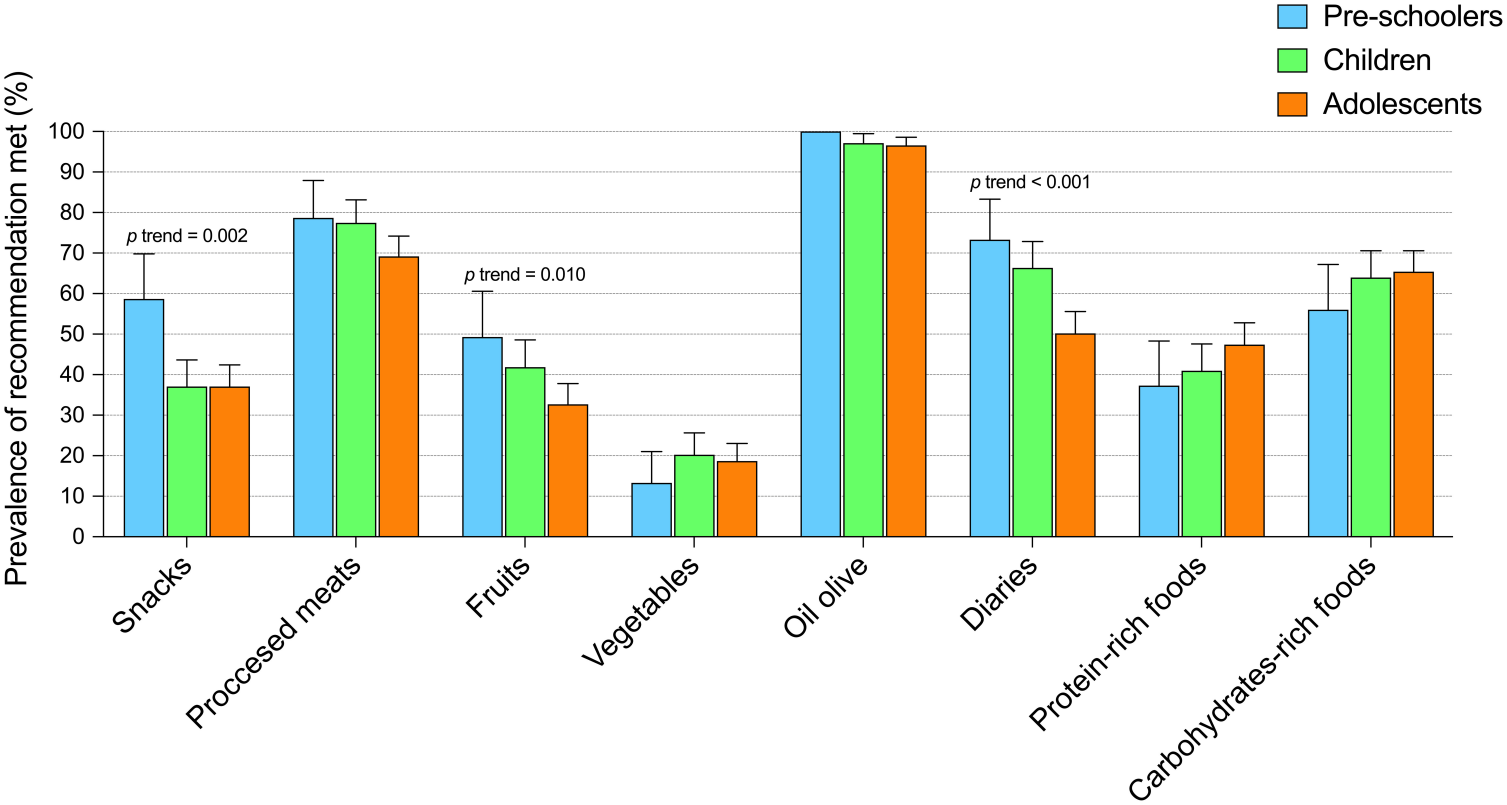
Prevalence of meeting of the Food-Based Dietary Guidelines stratified by age group.

Supplementary Table 2 depicts the food frequency during the COVID-19 lockdown among the Spanish young population. A percentage of 58.6% of participants ate vegetables/salads at least one time per day. The proportion of the young population who ate fruits at least one time per day was 10.5%. Regarding legumes, 65.6% of the participants never ate this type of food. Also, 19.5% indicated they never ate nuts and dried fruit. Figure 1 indicates the prevalence of adherence to the Food-Based Dietary Guidelines in the different age groups analyzed. An association between age group and the recommendations of snacks (*p* = 0.002), fruits (*p* = 0.010), and diaries (*p* < 0.001), was found.

**Figure 2:**
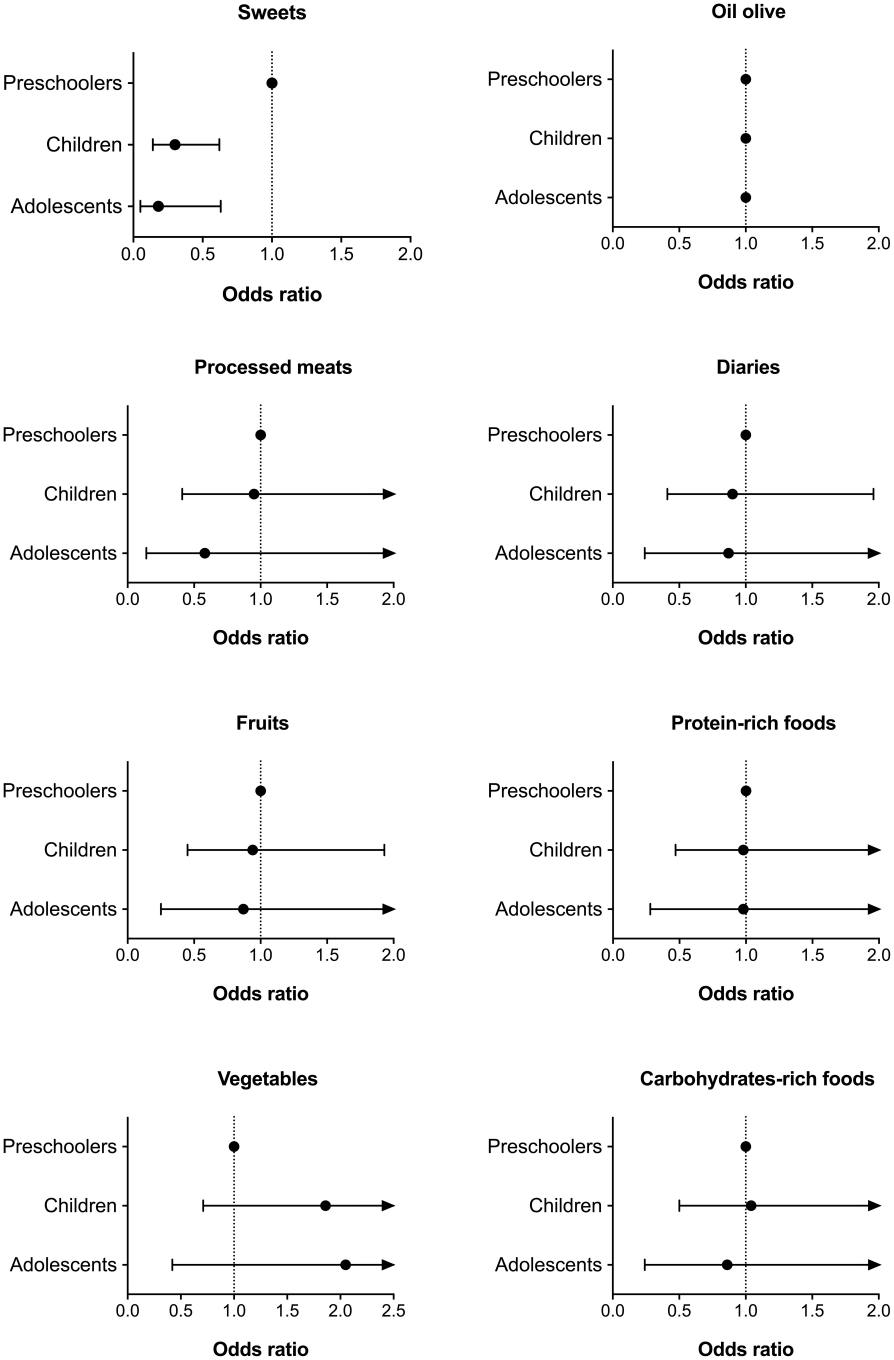
Association of meeting the different Food-Based Dietary Guidelines according to age group. Data expressed as odds ratio (confident intervals 95%). Adjusted by sex, socioeconomic status, educational level, geographical location, body mass index (z-score), and physical activity level.

Figure 2 shows the association between meeting individual Food-Based Dietary Guidelines in relation to age group, after adjusting for several covariates. A lower association with meeting the sweets recommendation was found in children (OR = 0.30; CI95%, 0.14–0.62), as well as in adolescents (OR = 0.18; CI95%, 0.05–0.63).

**Figure 3:**
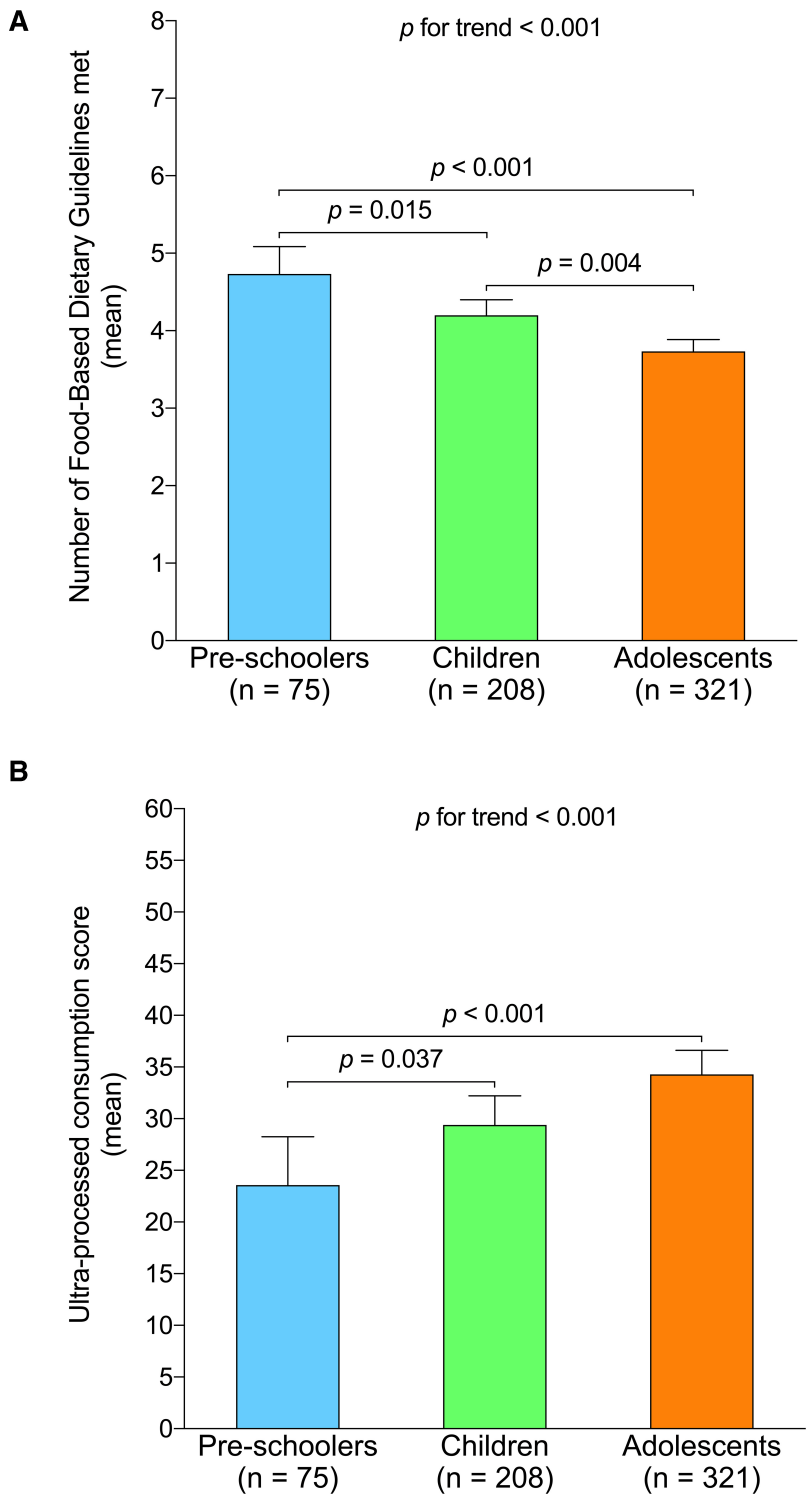
Mean differences of the number of Food-Based Dietary Guidelines met and ultra-processed consumption score according to age group. **(A)** Number of Food-Based Dietary Guidelines met. **(B)** Ultra-processed consumption score. Adjusted by sex, socioeconomic status, educational level, geographical location, body mass index (z-score), and physical activity level.

The mean differences of the number of Food-Based Dietary Guidelines met and ultra-processed consumption score according to age group are shown in the Figure 3. In relation to the Food-Based Dietary Guidelines (Figure 3A), adolescents showed a lower average of the meeting of these guidelines than both children (*p* = 0.004) and preschoolers (*p* < 0.001). Similarly, children reported a lower Food-Based Dietary Guidelines than preschoolers (*p* = 0.015). Regarding ultra-processed consumption (Figure 3B), we also observed a higher intake in adolescents than in children (*p* = 0.037), as well as in preschoolers (*p* < 0.001).

## Discussion

As far as we are concerned, this is the first study to report evidence of the adherence to Food-Based Dietary Guidelines and ultra-processed consumption during the COVID-19 lockdown among the Spanish young population. The current findings suggest low adherence to the Food-Based Dietary Guidelines among preschoolers, children, and adolescents during the COVID-19 lockdown, which is lower as age increases. Similarly, it has been pointed out a high ultra-processed consumption, mainly in adolescents.

In reference to dietary habits, the urgent need to improve them worldwide has recently been highlighted (4), since an inadequate diet constitutes a risk factor that causes more deaths than other factors, such as smoking (3). Food-Based Dietary Guidelines are helpful resources to clearly communicate an easy-to-understand information to a larger number of people, with the objective to aid the adherence of healthier eating habits (6, 7) based on the best available scientific evidence. However, as our findings suggested, most of the participants did not meet the national Food-Based Dietary Guidelines. Furthermore, a negative trend has been observed in older age groups, emphasizing the importance of encouraging the adherence to a healthy dietary pattern (and active lifestyle) during the early stages of life (24). In adults, the scientific evidence is not clear, with some studies showing an increase during the COVID-19 lockdown in healthy foods (11) and others in unhealthier foods (10). In young populations, the findings of the longitudinal study performed by Pietrobelli et al. (25) showed undesirable changes in lifestyle (e.g., eating healthy) in obese participants during the lockdown period. Likewise, one study that reported information after the COVID-19 lockdown in Spain showed a lower adherence to the Mediterranean diet [recognized as healthy dietary pattern (26)] among participants aged 11–16 (27). Conversely, one study among Spanish adolescents showed an increased consumption of fruit, as well as a decreased consumption of soft drinks, sweets and pastries, and convenience foods (28). However, these same authors found no statistically significant differences between groups. We hypothesize that the number of health-related behaviors of young populations (i.e., consumption of vegetables or fruits) as well as of their parents/legal guardians might have changed progressively during the COVID-19 lockdown, as one study in youths (29) has shown. In addition, another possible explanation is that the younger they are, the more responsibility parents/guardians have in making decisions about feeding. Nonetheless, caution is necessary to interpret these results, since we are not able to conclude that the findings obtained are exclusively favored by the COVID-19 lockdown.

Focusing on adherence to the Food-Based Dietary Guidelines at the individual level, the low consumption of fruits and, specially, vegetables among the young population was noteworthy. These foods are rich in micronutrients that are essential for the proper functioning of the immune system and have a vital influence on the promotion of health and nutritional well-being; they are even more necessary especially during the COVID-19 pandemic, as recommended by the WHO (8). One possible explanation could be related to work–life balance problems, as many parents/legal guardians had to telework while caring for their children during the closure of COVID-19. Thus, this scenario could lead to the adoption of less healthy eating habits, especially among those who are more dependent (e.g., young population) (29). Another possible justification could be the limited access to daily food shopping as a result of the COVID-19 lockdown, which may decrease the choice of fresh foods (e.g., fruits, vegetables), in favor of processed/ultra-processed foods, such as junk food or snacks, which tend to be higher in sugars, fats, or salt (30).

Regarding ultra-processed food, the results of a previous research with children and adolescents match with those achieved in the present study, highlighting the great influence of ultra-processed foods in the diet of adolescents. Ruíz-Roso et al. (31) showed that the habitual ultra-processed consumption was greater during the COVID-19 lockdown in their study performed in five different countries (Colombia, Chile, Brazil, Italy, and Spain). Notwithstanding, the differences on methodology to assess the consumption could influence the results obtained. There are some possible reasons for the high intake of ultra-processed foods during the COVID-19 lockdown. Firstly, the greater practicality characteristic of ultra-processed foods (i.e., durable, accessible, hyper-palatable) favors the intake of this unhealthier type of food (12), especially during periods of social isolation. Another possible reason is emotional eating, understood as the trend to overeat as a coping factor for controlling and decreasing undesirable feelings (e.g., stress, anxiety, depression) (32). In this sense, one study performed in Saudi Arabia (33) pointed out the usual influence of emotional eating in young females during the pandemic COVID-19, highlighting the importance of choosing healthy food during this health emergency situation.

Among the limitations of the present study, we declare the difficulty to compare our results with other studies because of the high variability of methods applied to research frequency food intake, as well as the lack of a specific score to determine the intake of ultra-processed food. Also, some types of food were not distinguished according to energy composition (e.g., cheese, chocolate), which made it difficult to categorize them into one group or another to establish compliance with the recommendations. There is no specific recommendation for the consumption of carbohydrate-rich foods according to the level of physical activity. However, the SENC advises that foods from this group should be consumed at each main meal. In this sense, a recent systematic review performed by Rabassa et al. (34) highlights the need to systematize, revise, and improve the development processes of Spanish Food-Based Dietary Guidelines. Accordingly, we tried to approximate the intake of carbohydrate-rich foods to this premise. Furthermore, the reliability on parent-reported data was also a limitation of this study. In addition, due to the cross-sectional nature of our study, it is not possible to determine causal inferences. Thus, future studies (mainly longitudinal and intervention designs) are required to report adherence to the Food-Based Dietary Guidelines and ultra-processed consumption to safeguard an adequate status during possible future scenarios of social isolation. Moreover, efforts should be directed toward parents influencing youths' eating behaviors by modeling their own eating behaviors, feeding practices they implement with their children, and their beliefs and attitudes about food (35). Also, responsible feeding and nutrition practices that support the child's autonomy to eat, as a response to their physiological requirements, which can promote self-regulation of feeding and assistance for young children's social, cognitive, and emotional development, should be encouraged (36). This fact is crucial at younger ages, since children acquire dietary habits from their personal experiences, but external experiences have a significant influence on preferences for what they prefer to eat (37, 38). In addition, we did not collect information on habitual dietary patterns before the COVID-19 lockdown. Therefore, we cannot infer whether the observations obtained changed during this scenario. Lastly, we used a snowball sampling to recruit participants. This approach, although prone to selection bias, is applicable in populations that are difficult to access because of their closed nature (39). Thus, due to the COVID-19 restrictions, this choice is justified.

The associations found highlight the low proportion of the young population meeting the Food-Based Dietary Guidelines and the high ultra-processed consumption during the COVID-19 lockdown. Similarly, there are important differences in meeting of Food-Based Dietary Guidelines and ultra-processed consumption among different age groups. The current results highlight the relevance of establishing public health policies and strategies for the young population, focusing on actions to encourage the adoption of healthy eating habits, particularly during and after phases of social isolation, with special emphasis in adolescents.

## Data Availability Statement

The raw data supporting the conclusions of this article will be made available by the authors, without undue reservation.

## Ethics Statement

The studies involving human participants this study was approved by the Ethical Committee of the Universidad Católica de Murcia (UCAM) (code: CE112001). Written informed consent to participate in this study was provided by the participants' legal guardian/next of kin.

## Author Contributions

JFL-G designed the study. JFL-G contributed to the interpretation of the data and to the analysis and writing of the draft. AG-H, JB-S, and PJT-L contributed to the revision of the manuscript. All authors approved the final version of the manuscript.

## Conflict of Interest

The authors declare that the research was conducted in the absence of any commercial or financial relationships that could be construed as a potential conflict of interest.

## Publisher's Note

All claims expressed in this article are solely those of the authors and do not necessarily represent those of their affiliated organizations, or those of the publisher, the editors and the reviewers. Any product that may be evaluated in this article, or claim that may be made by its manufacturer, is not guaranteed or endorsed by the publisher.

## References

[1] Ministerio de la Presidencia, Relaciones con las Cortes y Memoria Democrática. Real Decreto 463/2020, de 14 de marzo, por el que se declara el estado de alarma para la gestión de la situación de crisis sanitaria ocasionada por el COVID-19. (2021). Available online at: https://www.boe.es/eli/es/rd/2020/03/14/463/con (accessed April 25, 2021).

[2] Hossain MM Sultana A Purohit N. Mental health outcomes of quarantine and isolation for infection prevention: a systematic umbrella review of the global evidence. Epidemiol Health. (2020) 42:e2020038. 10.4178/epih.e2020038 32512661 PMC7644933

[3] Stanaway JD Afshin A Gakidou E Lim SS Abate D Abate KH. Global, regional, and national comparative risk assessment of 84 behavioural, environmental and occupational, and metabolic risks or clusters of risks for 195 countries and territories, 1990–2017: a systematic analysis for the global burden of disease study 2017. Lancet. (2018) 392:1923–94. 10.1016/S0140-6736(18)32225-6 30496105 PMC6227755

[4] Afshin A Sur PJ Fay KA Cornaby L Ferrara G Salama JS. Health effects of dietary risks in 195 countries, 1990–2017: a systematic analysis for the global burden of disease study 2017. Lancet. (2019) 393:1958–72. 10.1016/S0140-6736(19)30041-8 30954305 PMC6899507

[5] World Health Organization. Global Action Plan for the Prevention and Control of NCDs 2013–2020. Geneva, Switzerland: World Health Organization (2015). Available online at: http://www.who.int/nmh/events/ncd_action_plan/en/

[6] EFSA Panel on Dietetic Products Nutrition and Allergies (NDA). Scientific opinion on establishing food-based dietary guidelines. EFSA J. (2010) 8:1460. 10.2903/j.efsa.2010.1460

[7] World Health Organization ed. Preparation and use of food-based dietary guidelines: report of a Joint FAO/WHO Consultation. Geneva: WHO (1998). 9795598

[8] World Health Organization. Food and nutrition tips during self-quarantine. Geneva, Switzerland: World Health Organization (2020). Available online at: https://www.euro.who.int/en/health-topics/health-emergencies/coronavirus-covid-19/publications-and-technical-guidance/food-and-nutrition-tips-during-self-quarantine

[9] Ammar A Brach M Trabelsi K Chtourou H Boukhris O Masmoudi L. Effects of COVID-19 home confinement on eating behaviour and physical activity: results of the ECLB-COVID19 international online survey. Nutrients. (2020) 12:1583. 10.3390/nu12061583 32481594 PMC7352706

[10] Sánchez-Sánchez E Ramírez-Vargas G Avellaneda-López Y Orellana-Pecino JI García-Marín E Díaz-Jimenez J. Eating habits and physical activity of the spanish population during the COVID-19 pandemic period. Nutrients. (2020) 12:2826. 10.3390/nu12092826 32942695 PMC7551353

[11] Pérez-Rodrigo C Gianzo Citores M Hervás Bárbara G Ruiz Litago F Casis Sáenz L Aranceta-Bartrina J. Cambios en los hábitos alimentarios durante el periodo de confinamiento por la pandemia COVID-19 en España. Rev Esp Nutr Comunitaria. (2020) 26:101–11. 10.14642/RENC.2020.26.2.5213

[12] Monteiro CA Cannon G Moubarac J-C Levy RB Louzada MLC Jaime PC. The UN decade of nutrition, the nova food classification and the trouble with ultra-processing. Public Health Nutr. (2018) 21:5–17. 10.1017/S1368980017000234 28322183 PMC10261019

[13] Pagliai G Dinu M Madarena MP Bonaccio M Iacoviello L Sofi F. Consumption of ultra-processed foods and health status: a systematic review and meta-analysis. Br J Nutr. (2021) 125:308–18. 10.1017/S0007114520002688 32792031 PMC7844609

[14] Costa CS Del-Ponte B Assunção MCF Santos IS. Consumption of ultra-processed foods and body fat during childhood and adolescence: a systematic review. Public Health Nutr. (2018) 21:148–59. 10.1017/S1368980017001331 28676132 PMC10260745

[15] Lane MM Davis JA Beattie S Gómez-Donoso C Loughman A O'Neil A. Ultraprocessed food and chronic noncommunicable diseases: a systematic review and meta-analysis of 43 observational studies. Obes Rev. (2021) 22:e13146. 10.1111/obr.13146 33167080

[16] Skegg D Gluckman P Boulton G Hackmann H Karim SSA Piot P. Future scenarios for the COVID-19 pandemic. Lancet. (2021) 397:777–8. 10.1016/S0140-6736(21)00424-4 33607000 PMC7906624

[17] Schnohr CW Makransky G Kreiner S Torsheim T Hofmann F De Clercq B. Item response drift in the family affluence scale: a study on three consecutive surveys of the health behaviour in school-aged children (HBSC) survey. Measurement. (2013) 46:3119–26. 10.1016/j.measurement.2013.06.016

[18] Onis M Onyango AW Borghi E Siyam A Nishida C Siekmann J. Development of a WHO growth reference for school-aged children and adolescents. Bull World Health Organ. (2007) 85:660–7. 10.2471/BLT.07.043497 18026621 PMC2636412

[19] World Health Organization. WHO child growth standards: length/height-for-age, weight-for-age, weight-for-length, weight-for-height and body mass index-for-age; methods and development.De Onis M, editor. Geneva: WHO Press (2006).

[20] Subar AF Dodd KW Guenther PM Kipnis V Midthune D McDowell M. The food propensity questionnaire: concept, development, and validation for use as a covariate in a model to estimate usual food intake. J Am Diet Assoc. (2006) 106:1556–63. 10.1016/j.jada.2006.07.002 17000188

[21] Marcos Suarez V Rubio Mañas J Sanchidrián Fernández R Robledo de Dios T. Spanish national dietary survey on children and adolescents. EFSA Support Publ. (2015) 12:900. 10.2903/sp.efsa.2015.EN-900

[22] Aranceta-Bartrina J Partearroyo T López-Sobaler AM Ortega RM Varela-Moreiras G Serra-Majem L. Updating the food-based dietary guidelines for the spanish population: the spanish society of community nutrition (SENC) proposal. Nutrients. (2019) 11:2675. 10.3390/nu11112675 31694249 PMC6893611

[23] Prochaska JJ Sallis JF Long B. A physical activity screening measure for use with adolescents in primary care. Arch Pediatr Adolesc Med. (2001) 155:554. 10.1001/archpedi.155.5.554 11343497

[24] García-Hermoso A Ezzatvar Y López-Gil JF Ramírez-Vélez R Olloquequi J Izquierdo M. Is adherence to the mediterranean diet associated with healthy habits and physical fitness? A systematic review and meta-analysis including 565,421 youths. Br J Nutr. (2020) 10.1017/S0007114520004894. [Epub ahead of print]. 33292901

[25] Pietrobelli A Pecoraro L Ferruzzi A Heo M Faith M Zoller T. Effects of COVID-19 lockdown on lifestyle behaviors in children with obesity living in verona, Italy: a longitudinal study. Obesity. (2020) 28:1382–5. 10.1002/oby.22861 32352652 PMC7267384

[26] Galbete C Schwingshackl L Schwedhelm C Boeing H Schulze MB. Evaluating Mediterranean diet and risk of chronic disease in cohort studies: an umbrella review of meta-analyses. Eur J Epidemiol. (2018) 33:909–31. 10.1007/s10654-018-0427-3 30030684 PMC6153506

[27] Ventura PS Ortigoza AF Castillo Y Bosch Z Casals S Girbau C. Children's health habits and COVID-19 lockdown in catalonia: implications for obesity and non-communicable diseases. Nutrients. (2021) 13:1657. 10.3390/nu13051657 34068354 PMC8153273

[28] Aguilar-Martínez A Bosque-Prous M González-Casals H Colillas-Malet E Puigcorbé S Esquius L. Social inequalities in changes in diet in adolescents during confinement due to COVID-19 in Spain: the DESKcohort project. Nutrients. (2021) 13:1577. 10.3390/nu13051577 34066867 PMC8151229

[29] López-Bueno R López-Sánchez GF Casajús JA Calatayud J Gil-Salmerón A Grabovac I. Health-related behaviors among school-aged children and adolescents during the Spanish COVID-19 confinement. Front Pediatr. (2020) 8:573. 10.3389/fped.2020.00573 33042917 PMC7516648

[30] Di Renzo L Gualtieri P Pivari F Soldati L Attinà A Cinelli G. Eating habits and lifestyle changes during COVID-19 lockdown: an Italian survey. J Transl Med. (2020) 18:229. 10.1186/s12967-020-02399-5 32513197 PMC7278251

[31] Ruíz-Roso MB de Carvalho Padilha P Matilla-Escalante DC Brun P Ulloa N Acevedo-Correa D. Changes of physical activity and ultra-processed food consumption in adolescents from different countries during COVID-19 pandemic: an observational study. Nutrients. (2020) 12:2289. 10.3390/nu12082289 32751721 PMC7468997

[32] Ganley RM. Emotion and eating in obesity: a review of the literature. Int J Eat Disord. (1989) 8:343–61. 10.1002/1098-108X(198905)8:3<343::AID-EAT2260080310>3.0.CO

[33] Al-Musharaf S. Prevalence and predictors of emotional eating among healthy young saudi women during the COVID-19 pandemic. Nutrients. (2020) 12:2923. 10.3390/nu12102923 32987773 PMC7598723

[34] Rabassa M Hernández Ponce Y Garcia-Ribera S Johnston BC Salvador Castell G Manera M. Food-based dietary guidelines in Spain: an assessment of their methodological quality. Eur J Clin Nutr. (2021). 10.1038/s41430-021-00972-9 34282294

[35] Heller RL Mobley AR. Instruments assessing parental responsive feeding in children ages birth to 5 years: a systematic review. Appetite. (2019) 138:23–51. 10.1016/j.appet.2019.03.006 30853452

[36] Pérez-Escamilla R Segura-Pérez S Lott M. Feeding guidelines for infants and young toddlers: a responsive parenting approach. Nutr Today. (2017) 52:223–31. 10.1097/NT.0000000000000234

[37] Hoffmann DA Marx JM Kiefner-Burmeister A Musher-Eizenman DR. Influence of maternal feeding goals and practices on children's eating behaviors. Appetite. (2016) 107:21–7. 10.1016/j.appet.2016.07.014 27423817

[38] Kiefner-Burmeister AE Hoffmann DA Meers MR Koball AM Musher-Eizenman DR. Food consumption by young children: a function of parental feeding goals and practices. Appetite. (2014) 74:6–11. 10.1016/j.appet.2013.11.011 24275668

[39] Brewerton P Millward L. Organizational Research Methods. London, United Kingdom: SAGE Publications, Ltd. (2001). 10.4135/9781849209533

