# Supplementary material for: Dietary Patterns, Adherence to the Food-Based Dietary Guidelines, and Ultra-Processed Consumption During the COVID-19 Lockdown in a Sample of Spanish Young Population
Source: Front Pediatr. 2021 Oct 22;9:702731. doi: 10.3389/fped.2021.702731 (PMC8569821; doi:10.3389/fped.2021.702731)
Supplement: Supplementary file 1 [file Table_1.DOCX]

Additional file 1. Procedures to establish the adherence to the Spanish Society of Community Nutrition Food Based Guidelines among study participants.

| **Recommendation** | **Food item(s) selected** | **Method** | **Categorization** |
| --- | --- | --- | --- |
| **Occasionally, optional and moderated consumption** | | | |
| Level 1 (Sugary foods, pastries, spreads, salted snacks) | “Sweets”, “candies”, “industrial pastries”, “chips”, “chocolate”, “packaged milkshakes”, “sugary soft drinks”, “energy drinks”. | Sum of all foods from “sweets” category listed. The recommendation was considered met when there was no daily consumption of this type of food. | Re-coded as: Meeting (1) or Not-meeting (0) |
| Level 2 (Red meats, processed meats and sausages) | “Cold cuts”, “sausages”, “smoked sausages”, “industrial hamburger”. | Sum of all foods from “red/processed meats” category listed. The recommendation was considered met when there was no daily consumption of this type of food. | Re-coded as: Meeting (1) or Not-meeting (0) |
| **Daily varied consumption** | | | |
| Level 3A (Dairies) | “Milk”, “milk replacer enriched”, “cheese”, “yogurt”. | Sum of all foods from “diaries” listed. The recommendation was considered met when 2 or more servings of these foods were consumed daily. | Re-coded as: Meeting (1) or Not-meeting (0) |
| Level 3B (Fish, low fat meat, poultry, pulses, nuts, seeds, eggs) | “Smoked fish”, “canned fish”, “white fish”, “blue fish”, “fresh meat”, “wild game meat”, “chicken/turkey”, “eggs”, | Sum of all foods from “protein-rich foods” listed. The recommendation was met when 2 or more servings of these foods were consumed daily. | Re-coded as: Meeting (1) or Not-meeting (0) |
| Level 4A (Vegetables) | “Vegetable/salads”. | The recommendation was considered met when there was not a consumption of more than once a day of this type of food. | Re-coded as: Meeting (1) or Not-meeting (0) |
| Level 4B (Fruits) | “Fresh fruit or natural juice”, “dried fruit”. | Sum of all foods from “fruits” category listed. The recommendation was considered met when there was not a consumption of more than once a day of this type of food. | Re-coded as: Meeting (1) or Not-meeting (0) |
| Level 4C (Extra virgin oil olive) | “Uses olive oil at home”. | Specific question from the KIDMED questionnaire. | Re-coded as: Meeting (1) or Not-meeting (0) |
| Level 5 (Whole grain bread, whole grain pasta, whore grain rice, potatoes, chestnuts, tender legumes) | “Potatoes”, “white bread”, “whole wheat bread”, “breakfast cereals”. | Sum of all foods from “carbohydrates-rich foods” category listed. This recommendation was considered met when there was a consumption from 4 to 6 weekly (or higher) in at least three types of this food. | Re-coded as: Meeting (1) or Not-meeting (0) |
