# Supplementary material for: Dietary Patterns, Adherence to the Food-Based Dietary Guidelines, and Ultra-Processed Consumption During the COVID-19 Lockdown in a Sample of Spanish Young Population
Source: Front Pediatr. 2021 Oct 22;9:702731. doi: 10.3389/fped.2021.702731 (PMC8569821; doi:10.3389/fped.2021.702731)
Supplement: Supplementary file 2 [file Table_2.DOCX]

Additional file 2. Food frequency during the COVID-19 lockdown among Spanish young population (n = 604).

| **Food** | **Never** | **One to three times a month** | **Once a week** | **Two to three times a week** | **Four to six times a week** | **Once a day** | **More than once a day** |
| --- | --- | --- | --- | --- | --- | --- | --- |
| White bread | 47 (7.8) | 18 (3.0) | 33 (5.5) | 75 (12.4) | 83 (13.7) | 179 (29.6) | 169 (28.0) |
| Whole wheat bread | 302 (50.0) | 40 (6.6) | 48 (7.9) | 43 (7.1) | 50 (8.3) | 57 (9.4) | 64 (10.6) |
| **Breakfast cereals** | 189 (31.3) | 51 (8.4) | 45 (7.5) | 81 (13.4) | 54 (8.9) | 94 (15.6) | 90 (14.9) |
| **Industrial pastries** | 172 (28.5) | 113 (18.7) | 98 (16.2) | 88 (14.6) | 36 (6.0) | 47 (7.8) | 50 (8.3) |
| **Chips** | 110 (19.5) | 136(13.9) | 177(13.7) | 89(18.5) | 27(10.4) | 17(13.2) | 48(10.6) |
| **Salty snacks** | 163 (27.0) | 148 (24.5) | 128 (21.2) | 73 (12.1) | 24 (4.0) | 23 (3.8) | 45 (7.5) |
| Nuts and dried fruit | 118 (19.5) | 84 (13.9) | 83 (13.7) | 112 (18.5) | 63 (10.4) | 80 (13.2) | 64 (10.6) |
| Vegetables/Salad | 19 (3.1) | 20 (3.3) | 22 (3.6) | 75 (12.4) | 114 (18.9) | 159 (26.3) | 195 (32.3) |
| Legumes | 396 (65.6) | 112 (18.5) | 28 (4.6) | 15 (2.5) | 6 (1.0) | 8 (1.3) | 39 (6.5) |
| Potatoes | 418 (69.2) | 71 (11.8) | 37 (6.1) | 24 (4.0) | 7 (1.2) | 11 (1.8) | 36 (6.0) |
| Fresh fruit or natural juices | 25 (4.1) | 26 (4.3) | 81 (13.4) | 249 (41.2) | 160 (26.5) | 36 (6.0) | 27 (4.5) |
| **Fruit in syrup** | 396 (65.6) | 112 (18.5) | 28 (4.6) | 15 (2.5) | 6 (1.0) | 8 (1.3) | 39 (6.5) |
| Dried fruit (figs, raisins) | 418 (69.2) | 71 (11.8) | 37 (6.1) | 24 (4.0) | 7 (1.2) | 11 (1.8) | 36 (6.0) |
| Fresh meat (beef, pork, lamb, …) | 25 (4.1) | 26 (4.3) | 81 (13.4) | 249 (41.2) | 160 (26.5) | 36 (6.0) | 27 (4.5) |
| Wild game meat | 457 (75.7) | 56 (9.3) | 23 (3.8) | 17 (2.8) | 12 (2.0) | 5 (0.8) | 34 (5.6) |
| Chicken/turkey | 14 (2.3) | 13 (2.2) | 85 (14.1) | 268 (44.4) | 159 (26.3) | 44 (7.3) | 21 (3.5) |
| **Industrial hamburger** | 188 (31.1) | 210 (34.8) | 125 (20.7) | 31 (5.1) | 15 (2.5) | 8 (1.3) | 27 (4.5) |
| **Cold cuts (ham, turkey)** | 60 (9.9) | 48 (7.9) | 99 (16.4) | 177 (29.3) | 113 (18.7) | 67 (11.1) | 40 (6.6) |
| **Sausages (Sausage, Chorizo, ...)** | 142 (23.5) | 86 (14.2) | 98 (16.2) | 129 (21.4) | 75 (12.4) | 37 (6.1) | 37 (6.1) |
| **Smoked sausages other smoked sausages** | 220 (36.4) | 121 (20) | 128 (21.2) | 61 (10.1) | 34 (5.6) | 11 (1.8) | 29 (4.8) |
| Smoked fish (salmon, trout, ...) | 273 (45.2) | 99 (16.4) | 88 (14.6) | 76 (12.6) | 33 (5.5) | 11 (1.8) | 24 (4.0) |
| Canned fish (tuna, bonito, ...) | 113 (18.7) | 86 (14.2) | 150 (24.8) | 134 (22.2) | 65 (10.8) | 31 (5.1) | 25 (4.1) |
| White fish (hake, cod, sole, ...) | 80 (13.2) | 83 (13.7) | 196 (32.5) | 179 (29.6) | 41 (6.8) | 15 (2.5) | 10 (1.7) |
| Blue fish large (tuna, swordfish, ...) | 216 (35.8) | 148 (24.5) | 131 (21.7) | 62 (10.3) | 18 (3.0) | 13 (2.2) | 16 (2.6) |
| Blue fish small (sardine, anchovy, ...) | 236 (39.1) | 179 (29.6) | 108 (17.9) | 39 (6.5) | 14 (2.3) | 12 (2.0) | 16 (2.6) |
| Milk (whole, semi-skimmed, skimmed) | 92 (15.2) | 13 (2.2) | 12 (2.0) | 30 (5) | 69 (11.4) | 202 (33.4) | 186 (30.8) |
| **Milk replacer enriched** | 517 (85.6) | 19 (3.1) | 7 (1.2) | 5 (0.8) | 8 (1.3) | 11 (1.8) | 37 (6.1) |
| Plant milk (soy, oat, almond, …) | 422 (69.9) | 30 (5.0) | 16 (2.6) | 12 (2.0) | 33 (5.5) | 38 (6.3) | 53 (8.8) |
| Cheese | 55 (9.1) | 40 (6.6) | 69 (11.4) | 100 (16.6) | 124 (20.5) | 138 (22.8) | 78 (12.9) |
| Yogurt | 69 (11.4) | 37 (6.1) | 50 (8.3) | 97 (16.1) | 98 (16.2) | 175 (29) | 78 (12.9) |
| Eggs | 9 (1.5) | 17 (2.8) | 68 (11.3) | 237 (39.2) | 169 (28) | 75 (12.4) | 29 (4.8) |
| Sugar | 132 (21.9) | 60 (9.9) | 54 (8.9) | 82 (13.6) | 67 (11.1) | 129 (21.4) | 80 (13.2) |
| **Sweets/Candies** | 121 (20) | 136 (22.5) | 122 (20.2) | 103 (17.1) | 47 (7.8) | 26 (4.3) | 49 (8.1) |
| **Packaged milkshakes** | 315 (52.2) | 85 (14.1) | 66 (10.9) | 48 (7.9) | 31 (5.1) | 28 (4.6) | 31 (5.1) |
| **Industrial juices** | 291 (48.2) | 101 (16.7) | 46 (7.6) | 62 (10.3) | 37 (6.1) | 31 (5.1) | 36 (6.0) |
| **Isotonic drinks** | 362 (59.9) | 123 (20.4) | 38 (6.3) | 30 (5.0) | 17 (2.8) | 8 (1.3) | 26 (4.3) |
| **Energy drinks** | 505 (83.6) | 44 (7.3) | 13 (2.2) | 9 (1.5) | 6 (1.0) | 5 (0.8) | 22 (3.6) |
| **Sugary soft drinks** | 323 (53.5) | 107 (17.7) | 60 (9.9) | 43 (7.1) | 18 (3.0) | 19 (3.1) | 34 (5.6) |
| **Light soft drinks** | 417 (69.0) | 69 1(1.4) | 42 (7.0) | 21 (3.5) | 14 (2.3) | 11 (1.8) | 30 (5.0) |
| Coffee | 429 (71.0) | 41 (6.8) | 25 (4.1) | 29 (4.8) | 11 (1.8) | 36 (6.0) | 33 (5.5) |
| **Chocolate** | 82 (13.6) | 106 (17.5) | 124 (20.5) | 117 (19.4) | 85 1(4.1) | 58 (9.6) | 32 (5.3) |
| **Fitness food** | 484 (80.1) | 32 (5.3) | 16 (2.6) | 24 (4.0) | 17 (2.8) | 12 (2.0) | 19 (3.1) |
| **Industrial sauces (ketchup, mayonnaise, ...)** | 154 (25.5) | 104 (17.2) | 123 (20.4) | 84 1(3.9) | 62 1(0.3) | 38 (6.3) | 39 (6.5) |
| Table salt | 325 (53.8) | 63 1(0.4) | 51 (8.4) | 40 (6.6) | 37 (6.1) | 37 (6.1) | 51 (8.4) |

Data expressed as number (%). Bold indicates ultra-processed food according to the NOVA classification (12).
